# Implicit solvent effects on the binding interactions of amines with CO_2_

**DOI:** 10.1007/s00894-026-06863-9

**Published:** 2026-07-23

**Authors:** Jonathan de Brito Brum, José Walkimar de Mesquita Carneiro, Leonardo Moreira da Costa

**Affiliations:** Departamento de Química Inorgânica e Departamento de Química Orgânica, Instituto de Química, Programa de Pós-Graduação em Química, Universidade Federal Fluminense. Outeiro de São João Batista S/N, Niterói, RJ 24020-141 Brazil

**Keywords:** Amine-based solvent, CO_2_ capture and storage, DFT, Implicit solvation, Interaction analysis

## Abstract

**Context:**

CO_2_ capture and storage using amine-based solvents is a widely explored strategy in the literature aimed at mitigating the environmental impact associated with large-scale fossil fuel combustion. In the present work, four amines with distinct basicity levels were modeled in fifteen solvents with dielectric constants ranging from 1.88 (hexane) to 111 (formamide), in order to assess which implicit solvation approach, PCM, CPCM, or SMD, provides co-solvation results in closer agreement with theoretical and experimental data reported in the literature. A comprehensive analysis of implicit solvation effects was conducted, examining both the structural consequences of CO_2_ capture and the variations in stabilization energies associated with the formation of the zwitterionic intermediate. The results indicate that the SMD solvation model exhibits trends more consistent with literature data, owing to its sensitivity to local solute–solvent interactions, particularly hydrogen bonding. Notably, the SMD parameterization incorporates hydrogen-bond acidity and basicity descriptors derived from the Abraham solvation model, enabling correlation analyses between these parameters and the thermodynamic and solvation quantities. These findings provide deeper insight into the fundamental role of co-solvation in CO_2_ capture by amine-based solvents, particularly in reducing the free energy of stabilization of the zwitterionic state. Furthermore, this study identifies the implicit solvation approach that, when combined with DFT, yields result most consistent with established theoretical and experimental benchmarks reported in the literature.

**Method:**

All calculations were performed at the DFT CAM-B3LYP/6–311++G(d,p) level of theory.

**Supplementary Information:**

The online version contains supplementary material available at 10.1007/s00894-026-06863-9.

## Introduction

Over the past century, accelerated industrial growth and urban development have contributed to substantial economic expansion and an increase in global population [1, 2]. Consequently, worldwide energy demand has grown significantly, with fossil fuels being the primary source for production chains [3]. The combustion of fossil fuels substantially enhances CO_2_ emissions, which are associated with rising global temperature, climate change, respiratory diseases, and ocean acidification, potentially resulting in extensive species extinction [4, 5]. This context demands the implementation of immediate, scalable and technically viable interventions to mitigate further environmental degradation and to facilitate the transition toward a sustainable, low-carbon energy system. Carbon Capture, Utilization and Storage (CCUS) technologies are becoming essential strategies for mitigating CO_2_ emissions by developing sorbent materials, high performance membranes, and electrochemical conversion systems [6, 7]. The development of chemisorbent materials such as zeolites, metal organic frameworks (MOFs), microporous polymer networks, and amine-functionalized systems for CO_2_ sequestration is a promising strategy to reduce atmospheric carbon levels [7–10]. These techniques can effectively capture CO_2_ at major emission sources, including power generation facilities and energy-intensive industrial plants, thereby preventing its direct release into the atmosphere [11].

Computational studies [12–15] on the design of the amine chemisorbents have achieved substantial progress for the rationalization and optimization of amine-CO_2_ interactions. Zhou et al*.* [12] performed B3LYP/6–311++G(d,p) calculations with the SMD solvation model to assess how the steric architecture of amines affects CO_2_ interaction strength and absorption kinetics. They found that increased molecular steric hindrance reduces the nucleophilicity of amine nitrogen atoms. Additionally, ethanol’s solvation effect reduces the amine reactivity toward CO_2_, resulting in lower absorption rates in ethanol-based systems compared to aqueous environments. Higashii et al. [13] conducted a computational investigation of CO_2_ absorption in aqueous alkanolamine solutions employing B3LYP/6–311++G(d,p) with the polarizable continuum solvation model (PCM). This work systematically explored the influence of alkyl chain length and hydroxyl groups position on the reactivity of alkanolamines toward CO_2_, demonstrating that the position of the hydroxyl group impacts reaction product formation through intramolecular hydrogen-bonding interactions; variations in alkyl chain length have only a minor influence on the overall process. Chi et al. [14] applied the B3LYP/6–311 + G(d,p) DFT method and the SMD continuum solvation model to investigate the reaction pathway between arginine and alanine amino acids and CO_2_. Results show that alanine does not exhibit spontaneous chemical absorption, whereas arginine forms thermodynamically favorable (∆G < 0) adducts due to its capacity to promote intra- and intermolecular proton transfer to the CO_2_ molecule, resulting in carbamic acid and carbamate formation. Yamada et al*.* [15] calculated pKa values for 25 amines (alkanolamines, cyclic, and aromatic) using the COSMO-RS [16] and the SM5.4/A [17] solvation methods coupled with the density functional theory. The predictions of pKa values were compared using different DFT levels (BP/TZVP, B3LYP/6–311++G(d,p) and B3LYP/6-31G(d)) for geometry optimization. The BP/TZVP level combined with COSMO gave the highest correlation with experimental values for the 25 amines (R^2^ = 0.8). Additional theoretical studies [18–23] examining amine-CO_2_ interactions have corroborated these trends. In previous studies [24–27], our group demonstrated that amine-CO_2_ interactions induces a structural change in CO_2_ from a linear to a bent geometry within the zwitterionic intermediate. Furthermore, the interaction energy magnitude is directly related to the intrinsic basicity of the amine, with stronger bases promoting more favorable and energetically stable interactions.

The adoption of a specific solvation model in computational studies is a critical element according to recent studies. Methods based on the Polarizable Continuum Model (PCM) [28, 29], including the Conductor-like Polarizable Continuum Model (CPCM) [30], as well as the density-based universal solvation model (SMD) [31], have been employed to achieve closer agreement with experimental results. Within the PCM framework, a cavity is constructed around the solute, with the surrounding solvent represented as a polarized dielectric continuum characterized by its dielectric constant (ε). Solute electron density induces apparent surface charges on the cavity boundary; these charges are subsequently determined and incorporated into the final Hamiltonian as a perturbative term, allowing the assessment of solvation effects [28, 29]. The CPCM approach follows a similar formalism, initially modeling the solvent as a perfect conductor and then scaling the resulting polarization to reproduce the dielectric properties of the target solvent [28–30]. By contrast, the SMD model incorporates an alternative conceptual approach. It defines solvation free energy as a combination of electrostatic contributions from the polarized continuum and cavity-dispersion-solvent structure (CDS) terms. The CDS contributions are parameterized in terms of atomic surface tensions, solvent-accessible surface areas, acidity and basicity descriptors, van der Waals radii, and related quantities, aiming to reproduce the local interactions at the solute–solvent interface [31].

**Fig. 1:**
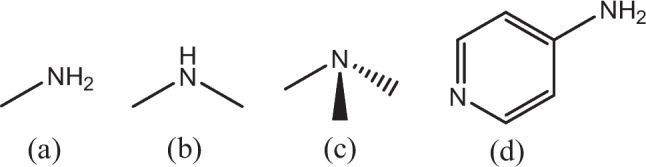
Chemical structures of selected amines along with their respective abbreviations: (**a**) methylamine (MeNH_2_); (**b**) dimethylamine (Me_2_NH); (**c**) trimethylamine (Met_3_N); (**d**) pyridin-4-amine (PyNH_2_)

Several studies reported in the literature emphasize the significant influence of hydrogen bonding on the CO_2_ capture process, underscoring the necessity of employing a solvation model that can accurately represent these interactions [32, 33]. Optimization of amine-CO_2_ adducts in the gas phase generally yields structures characterized by great amine-CO_2_ distances and an almost linear CO_2_ geometry, indicating limited acid–base interaction. Consequently, a comprehensive evaluation of adduct geometries using different solvation approaches is worth. Accordingly, we calculated the interactions between CO_2_ and four representative amines (Fig. 1) to determine how the different solvation approaches behave toward the energetic of the amine-CO_2_ interaction process. This analysis provides valuable insights that can guides the design of amine ligands with improved properties in condensed phase for more effective CO_2_ capture.

## Computational Details

**Fig. 2:**
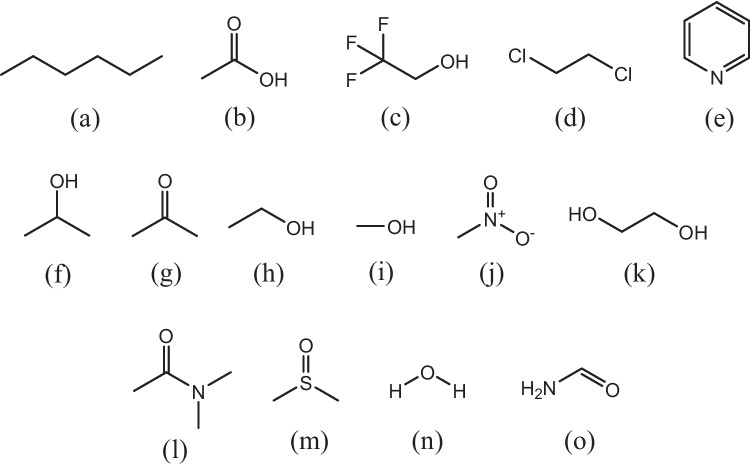
The solvents employed: (**a**) hexane (ε = 1.88); (**b**) acetic acid (ε = 6.15); (**c**) 2,2,2-trifluoroethanol (ε = 8.55); (**d**) 1,2-dichloroethane (ε = 10.36); (**e**) pyridine (ε = 12.4); (**f**) isopropanol (ε = 17.9); (**g**) acetone (ε = 20.7); (**h**) ethanol (ε = 24.50); (**i**) methanol (ε = 32.70); (**j**) nitromethane (ε = 35.87); (**k**) ethylene glycol (ε = 37.0); (**l**) N, N-dimethylacetamide (ε = 37.8); (**m**) dimethyl sulfoxide (ε = 46.7); (**n**) water (ε = 80.1); (**o**) formamide (ε = 111.0)

Geometry optimizations and vibrational frequency analysis were conducted using the Gaussian 16 [34] and ORCA 6.0 [35, 36] programs, employing the CAM-B3LYP functional [37] with the 6–311++G(d,p) basis set [38]. Previous studies by our group have shown that this computational approach yields results consistent with higher level calculation methods [24]. To incorporate solvent effects, three implicit solvation models, PCM [28, 29], CPCM [30], and SMD [31] were employed, using solvents with dielectric constants ranging from 1.88 to 111 (see Fig. 2). The implicit solvents considered included formamide (FMA), water, dimethyl sulfoxide (DMSO), N,N-dimethylacetamide (DMAc), ethylene glycol, nitromethane (MetNO2), methanol (MetOH), ethanol (EtOH), acetone, isopropanol, pyridine (Py), 1,2-dichloroethane, 2,2,2-trifluoroethanol (TFE), acetic acid, and hexane. Frequency calculations at the same theoretical level were carried out to verify that the stationary points represent true minimum on the potential energy surface, and to provide thermodynamic corrections for enthalpy and Gibbs free energy under standard conditions (1 atm and 298 K).

## Results and Discussion

### Geometry Optimization

**Fig. 3:**
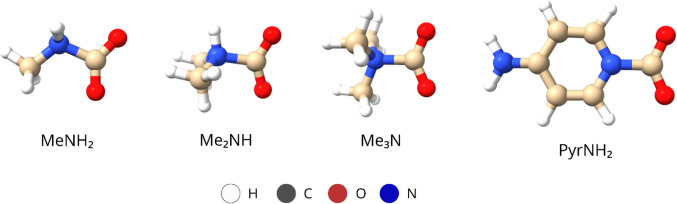
Optimized geometries of the amine-CO_2_ adducts in implicit water (SMD solvation model)

The geometries of 176 amine-CO_2_ adducts were fully optimized using the CAM-B3LYP/6–311 + + G(d,p) computational method, comprising four adducts evaluated across fifteen solvents with three solvation models. Figure 3 shows examples of final adducts, as obtained by geometry optimizations in water employing the SMD solvation model.

The analysis of Fig. 3 shows that, in all adduct structures for these examples, the geometry of the CO_2_ becomes angular, in contrast to the linear arrangement of the free molecule. This is attributed to the charge transfer from the amines to the carbon of CO_2_ via inductive effects, which modifies the carbon hybridization, resulting in variation of the OCO bond angle. Additionally, steric repulsion from amines with larger aliphatic chains further modifies the OCO angle, thus affecting the acid–base interaction [39]. In the optimized geometries, each ligand coordinates to the CO_2_ carbon atom through a single binding site (monodentate). However, such coordination occurs only due to the stabilization provided by the external solvent; in environments with zero or very low dielectric constant (such as vacuum or hexane), the adduct lacks sufficient charge stabilization in the zwitterionic state, thus the N–C bond does not form, thereby preventing effective CO2 capture.

**Fig. 4:**
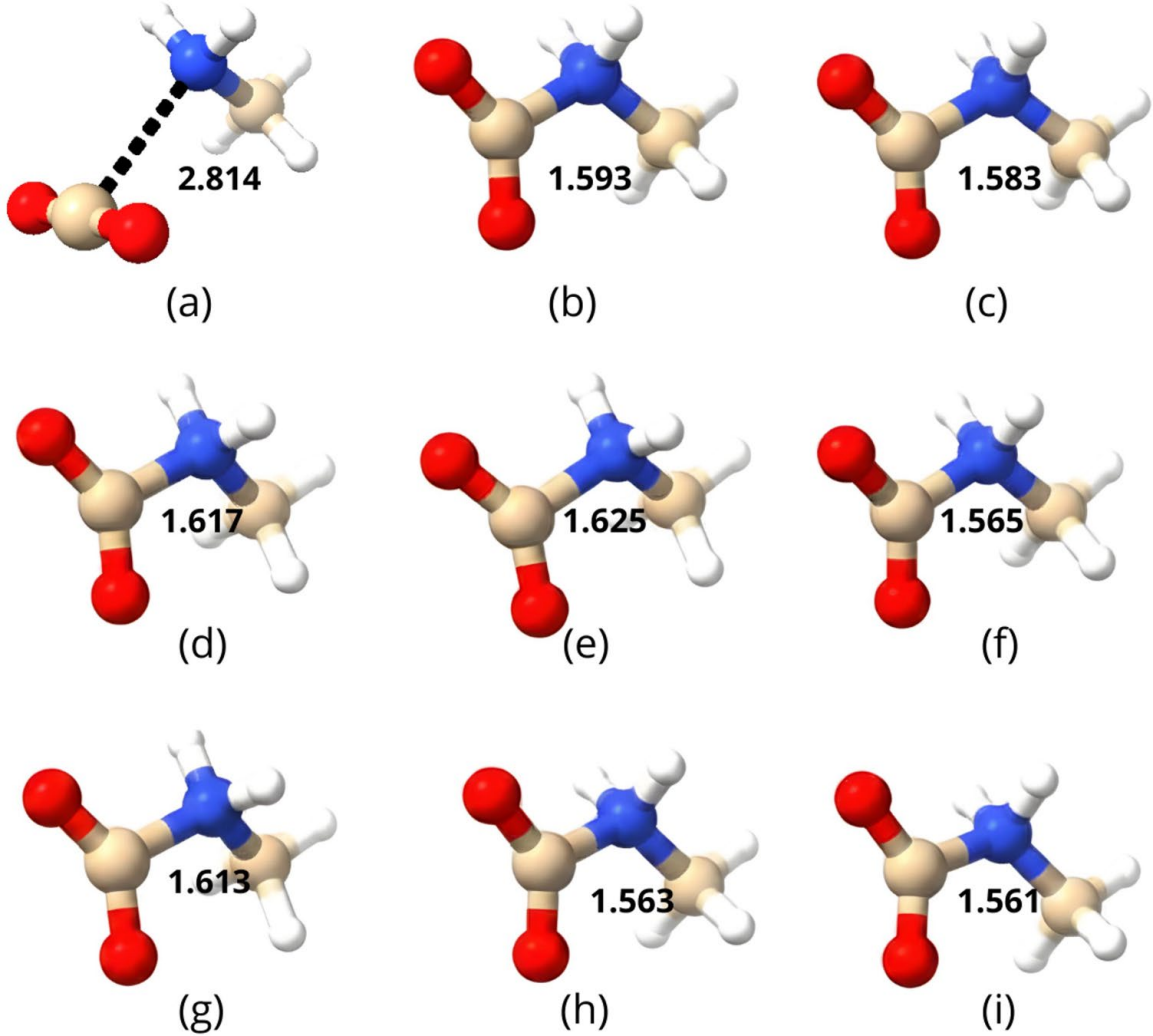
Optimized geometries of methylamine-CO_2_ adducts, in implicit solvent using the SMD model. The given values are the N–C distance (in Å) in the optimized geometries. Solvents and their corresponding dielectric constants are as follows: (**a**) Hexane (ε = 1.88); (**b**) Acetic acid (ε = 6.15); (**c**) Trifluoroethanol (ε = 8.55); (**d**) 1,2-Dichloroethane (ε = 10.36); (**e**) Pyridine (ε = 12.40); (**f**) Ethylene glycol (ε = 37.00); (**g**) N,N-Dimethylacetamide (ε = 37.80); (**h**) Water (ε = 80.01); (**i**) Formamide (ε = 111.00)

Figure 4 shows the optimized geometries of the methylamine adduct obtained in selected solvents spanning a wide range of dielectric constants. Detailed geometric parameters, such as bond lengths and the OCO angle, are provided in Section S1 of the Supporting Information.

Figure 4 illustrates the significant challenges associated with bond formation in nonpolar environments, such as hexane; similar behavior is also observed in vacuum, hydrocarbons, and low-polarity solvents, where the nitrogen-carbon bond indeed does not form. This trend is consistent with the literature: Wang et al. [33] reported that zwitterionic states are not stable in chloroform and propylamine due to their low dielectric constants, which may be attributed to the lack of external stabilization of the charges generated in the zwitterionic state, hindering the stabilization of the adduct. Additionally, small variations in bond distances are observed as a function of the solvent employed. Acetic acid (1.593 Å) and trifluoroethanol (1.583 Å), despite their relatively low dielectric constants, exhibit slightly shorter bond lengths compared to solvents such as pyridine (1.625 Å) and N,N-dimethylacetamide (1.613 Å), which have higher dielectric constants. A similar trend is observed between N,N-dimethylacetamide and ethylene glycol, whose dielectric constants area nearly identical (ε ≈ 37); nevertheless, the bond length is shorter in ethylene glycol (1.565 Å) than in N,N-dimethylacetamide (1.613 Å). This behavior is attributed to the presence of hydrogen bonding in the solvents acetic acid, trifluoroethanol and ethylene glycol, which provides additional stabilization to the zwitterionic system. Ethylene glycol has been investigated for its co-solvation effects in amine-based solvents, with the literature reporting an enhanced CO_2_ absorption capacity in solution and more negative absorption enthalpy when compared to aqueous amine solutions [40, 41]. Solvents with hydrogen-bonding capability exhibit reduced bond lengths, reflecting increased stabilization of the adduct. When both high polarity and hydrogen-bonding capability are present in a solvent, such as in water and in formamide, the system achieves superior stabilization, showing that both effects act synergistically to enhance CO_2_ capture. Table 1 presents the bond distances and bond angles for methylamine and 4-aminopyridine in hexane, acetic acid, trifluoroethanol, dimethyl sulfoxide, water, and formamide, as obtained in the three implicit solvation models. The complete set of geometric parameters, including bond distances and the OCO angle, are provided in Section S1 of the Supporting Information.

**Table 1:** Selected interaction distances (Å) between the NH_2_ amino group and CO_2_ calculated for hexane, acetic acid, trifluoroethanol, dimethyl sulfoxide, water, and formamide using the PCM, CPCM, and SMD solvation models

Solvent	PCM		CPCM		SMD	
	MetNH_2_	PyrNH_2_	MetNH_2_	PyrNH_2_	MetNH_2_	PyrNH_2_
Hexane (ε = 1.88)	2.813	1.701	2.834	1.654	2.814	1.665
Acetic (ε = 6.15)	1.708	1.584	1.636	1.564	1.593	1.535
2,2,2-trifluoroethanol (ε = 8.55)	1.649	1.559	1.623	1.556	1.583	1.528
DMSO (ε = 46.70)	1.642	1.556	1.601	1.540	1.612	1.560
Water (ε = 80.01)	1.639	1.554	1.599	1.539	1.563	1.510
Formamide (ε = 111.00)	1.644	1.554	1.599	1.538	1.561	1.509

Table 1 shows a correlation between the dielectric constant of the solvent and the interaction distance between the nitrogen atom of the amine and CO_2_ with the PCM and CPCM models: geometries optimized in solvents with higher dielectric constants have shorter nitrogen-carbon distances. This trend is absent in the SMD model. As an example, PCM results for DMSO show that MetNH_2_ (1.642 Å) and PyrNH_2_ (1.556 Å) exhibit shorter interaction distances compared to solvents with lower dielectric constant, such as acetic acid, where MetNH_2_ (1.708 Å) and PyrNH_2_ (1.584 Å) have increased distance between the interaction sites. Conversely, the SMD model presents the opposite pattern: despite its lower dielectric constant, acetic acid exhibits shorter interaction distances, MetNH_2_ (1.593 Å) and PyrNH_2_ (1.535 Å), than those obtained with DMSO, MetNH_2_ (1.612 Å) and PyrNH_2_ (1.560 Å). This behavior is attributed to DMSO’s lack of hydrogen-bond donor and acceptor sites, which reduces adduct stabilization, despite its high dielectric constant. The SMD model explicitly incorporates hydrogen bonding effects through Abraham’s α and β parameters [42–44], representing hydrogen-bond acidity and basicity, respectively. In contrast, classical implicit solvation models such as PCM and CPCM primarily depend on dielectric constant and solvent polarity, without considering hydrogen bonding contributions.

### Thermochemistry

**Fig. 5:**
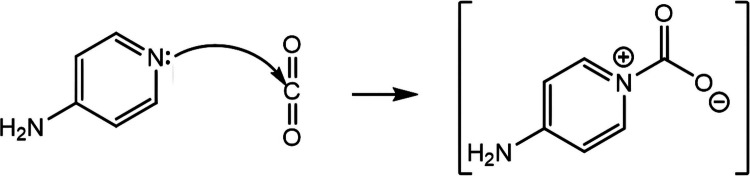
Schematic illustration of the CO_2_ capture process using 4-aminopyridine as a representative amine

The thermodynamic properties were calculated considering the reaction for CO_2_ capture, which is driven by electron density donation from the amino group to the CO_2_ molecule. We consider the energy difference between the resulting zwitterionic state and the isolated amine and CO_2_ molecules. Figure 5 provides a schematic representation of the CO_2_ capture process by amine-based solvents.

**Fig. 6:**
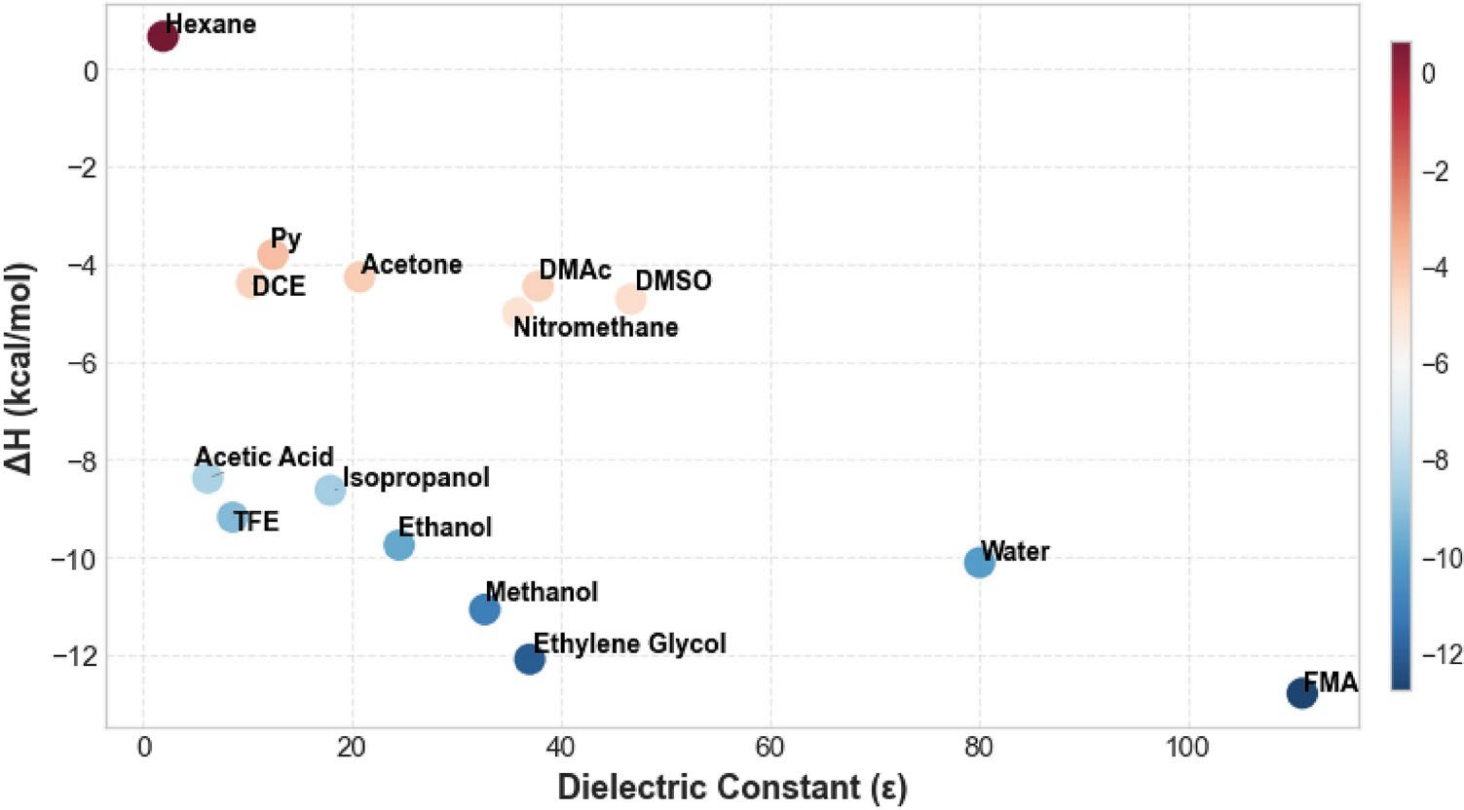
Relative stabilities of the 4-aminopyridine zwitterionic intermediate (ΔH) as a function of the dielectric constant of the solvents, using the SMD model

Table 2 and Fig. 6 display the variation in Gibbs free energy and the relative stability of the adduct as a function of the dielectric constant of the implicit solvent, using the SMD model for CO_2_ capture by 4-aminopyridine. The thermodynamic parameters, including enthalpy and Gibbs free energy changes for all solvents and solvation models, are provided in Section S2 of the Supporting Information.

**Table 2:** Enthalpy and Gibbs free energy changes for the formation of the zwitterionic state of 4-aminopyridine, calculated for fifteen solvents using the SMD solvation model

Solvent	PCM		CPCM		SMD	
	ΔH	ΔG	ΔH	ΔG	ΔH	ΔG
Hexane	1.81	11.49	−2.02	6.77	0.66	11.50
Acetic Acid	−1.24	8.94	−2.86	8.50	−8.38	3.15
2,2,2-trifluoroethanol	−2.99	7.33	−3.57	7.83	−9.18	2.39
1,2-Dichloroethane	−2.07	8.18	−3.90	7.52	−4.39	7.07
Pyridine	−2.39	7.89	−4.16	7.26	−3.81	7.64
Isopropanol	−2.77	7.54	−4.58	6.87	−8.63	2.95
Acetone	−2.82	7.50	−4.71	6.74	−4.27	7.22
Ethanol	−2.95	7.37	−4.84	6.62	−9.75	1.83
Methanol	−3.10	7.23	−5.02	6.45	−11.07	0.56
Nitromethane	−3.15	7.18	−5.07	6.40	−5.00	6.52
Ethylene Glycol	–-	–-	−5.09	6.38	−12.09	−0.46
N,N-dimethylacetamide	−3.17	7.17	−5.10	6.38	−4.46	7.03
DMSO	−3.25	7.09	−5.19	6.29	−4.72	6.78
Water	−3.39	6.96	−5.35	6.13	−10.11	1.48
Formamide	−3.45	6.90	−5.41	6.08	−12.79	−1.14

The thermodynamic properties for the adduct formation are impacted by the solvent, as are the geometric parameters, according to the previous discussion. Stabilization values vary between approximately −5.00 (Nitromethane) to −8.38 (Acetic Acid) kcal/mol, corresponding to a ΔH gap of 3.38 kcal/mol. A gap of roughly 4 kcal/mol suggests hydrogen bonds of moderate strength [45, 46], indicating that the SMD model effectively captures these interactions. Furthermore, protic and aprotic solvents display differing degrees of stabilization, largely governed by the dielectric constant of each class of solvent. Accordingly, solvents such as water and ethylene glycol exhibit the most negative interaction enthalpy, in agreement with both theorical and experimental data reported in the literature [20, 40, 41].

**Fig. 7:**
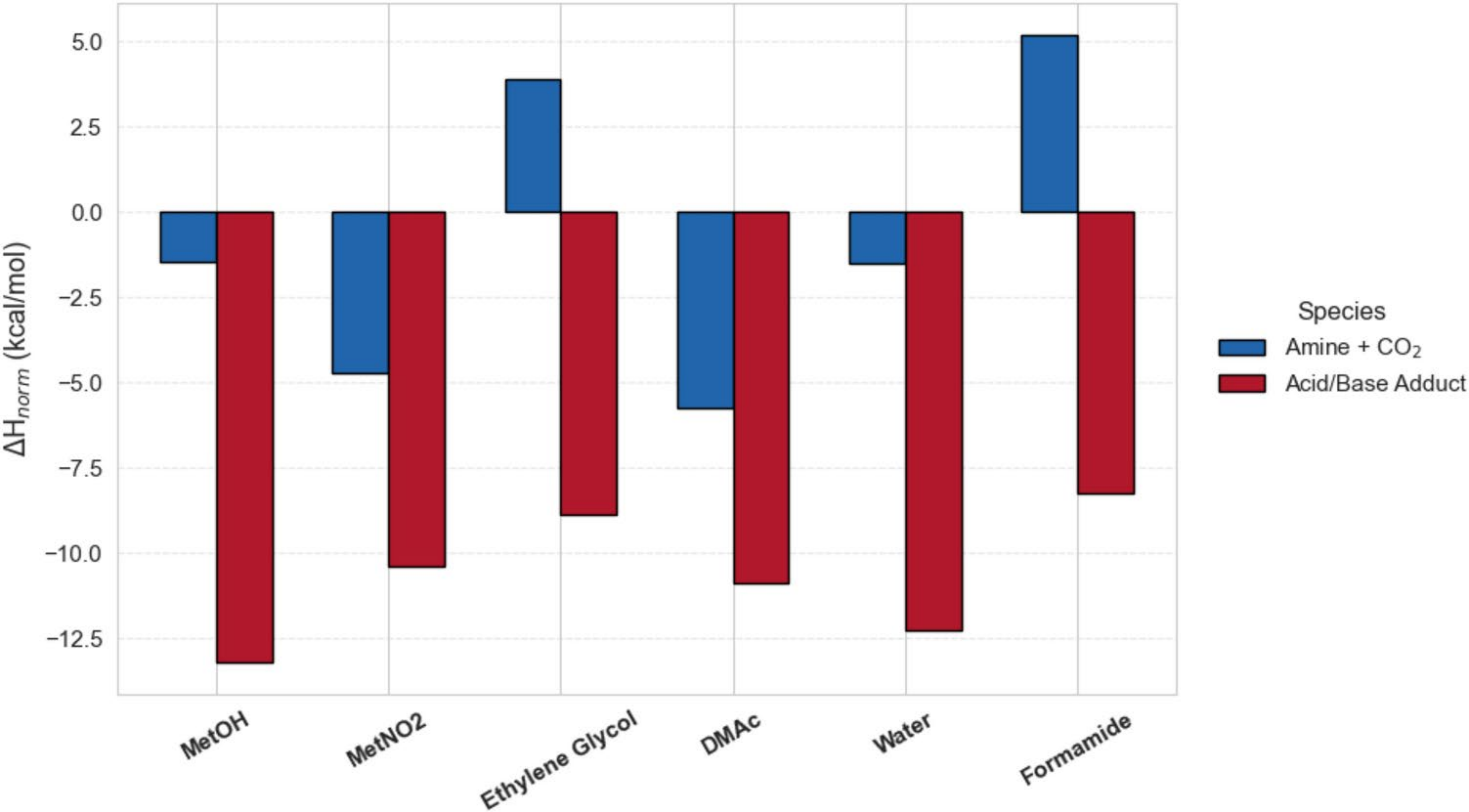
Solvation energies of the reactants and the 4-aminopyridine zwitterionic intermediate in different solvents, according to the SMD model. The solvation energy in hexane is taken as 0 kcal/mol as the reference

To rationalize the differential stabilization of the amine-CO_2_ adduct in the different solvents, we computed the individual solvation energies of the isolated reactants (amine and CO_2_) and the zwitterionic adduct. Figure 7 shows the result for 4-aminopyridine using the SMD model. For the calculation of the relative stabilization between the isolated reactants and the zwitterionic form, the value obtained in hexane was used as a reference, given the difficulty in forming zwitterionic states in the gas phase. The relative stabilization values for the remaining molecules, solvents, and solvation models are provided in Section S3 of the Supporting Information.

**Fig. 8:**
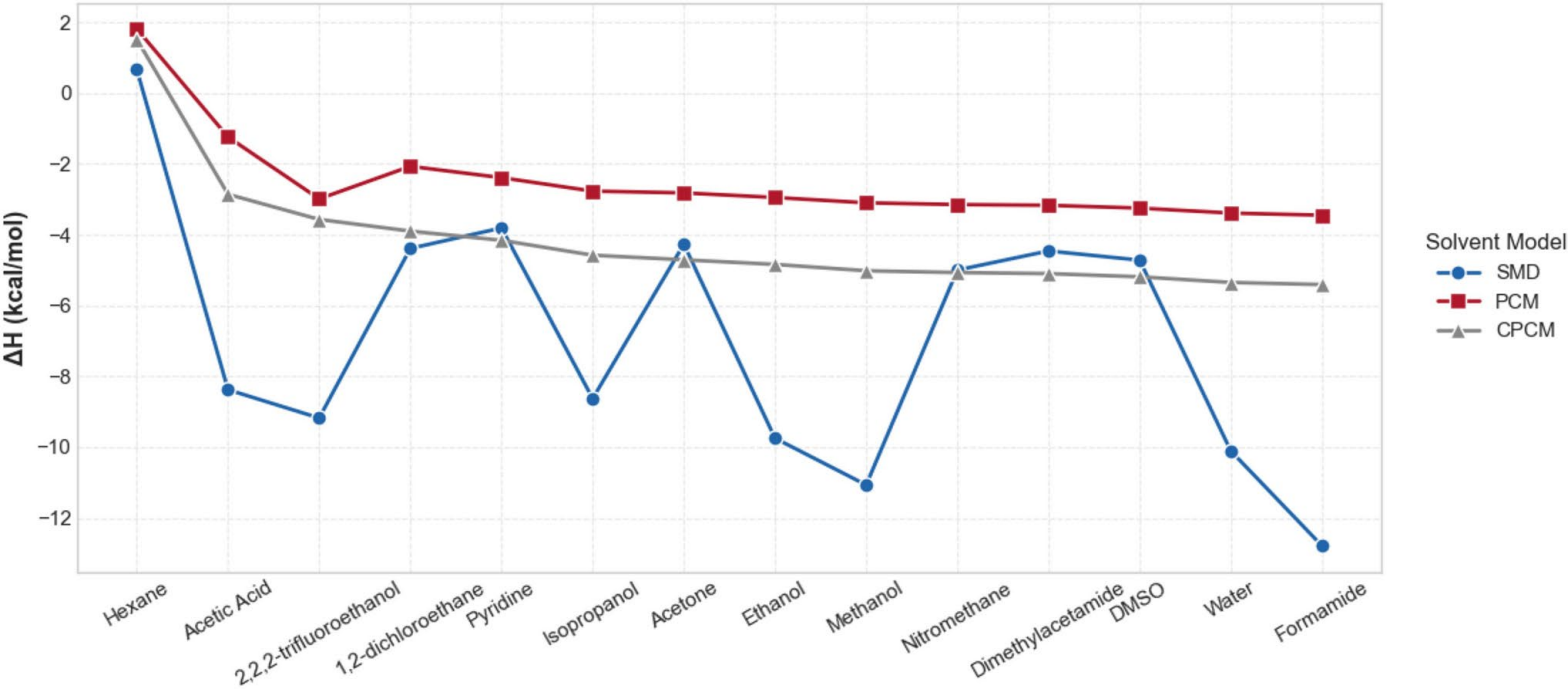
Relative stabilities (ΔG) of the dimethylamine zwitterionic intermediate across different implicit solvation models

All solvents shown in Fig. 7 stabilize the zwitterionic state relative to the calculation in hexane, as evidenced by their markedly negative solvation energies of the zwitterionic state. The same behavior was observed for the isolated species, except for the protic solvents ethylene glycol (+ 3.89 kcal/mol) and formamide (+ 5.22 kcal/mol), for which the isolated species experience a slight destabilization, indicating that the electrostatic component of the solvation model is outweighed by the cavitation term, resulting in a positive total solvation energy of the isolated species. This destabilization of the reactants contributes to highly negative variations in Gibbs free energy of the adsorption process in these two solvents. Among the polar solvents, water (−1.48 kcal/mol) and methanol (−1.46 kcal/mol) provide weak stabilization for the isolated species. Thus, the reaction is favored only when there is strong stabilization of the acid–base adduct. This effect is observed exclusively in solvation models that explicitly account for hydrogen-bonding interactions; other implicit solvation approaches do not reproduce this phenomenon, as can be observed in Fig. 8, which presents the relative stabilization energies of the dimethylamine zwitterionic state as a function of the solvent for each of the three solvation models considered.

The PCM and CPCM models exhibit only minor variations in stabilization when the implicit solvent is altered, in contrast to the SMD model, which shows a more pronounced variation and, in several cases, shows an opposite trend. For example, comparing DMSO with ethanol, PCM and CPCM both predict higher stabilization of the zwitterionic state in DMSO, 0.27 kcal/mol e 0.34 kcal/mol respectively, whereas the SMD model yields greater stabilization in ethanol (4 kcal/mol). This behavior is also observed for the other protic solvents water, methanol, acetic acid, and N,N-dimethylacetamide, once again demonstrating that classical solvation models mainly account for solvent polarization and fail to capture the depth of relevant intermolecular hydrogen-bonding interactions. Notably, exceptions arise when polarity contributions are particularly significant, such as with formamide and hexane, which represent solvents with the highest and lowest dielectric constants, respectively.

### Hydrogen-bond correlation

The Abraham general solvation model [47, 48] employs several physicochemical parameters to describe the solubility of a solute in a specific organic solvent. Based on the linear free energy relationship (LFER) [48], the Abraham model uses Eq. 1 to calculate the molar concentration of the solute in the organic solvent (*Ss*).

1 $$\mathrm{log}{S}_{s}=\mathrm{log}{S}_{w}+c + eE+sS+aA+bB + v V$$

**Fig. 9:**
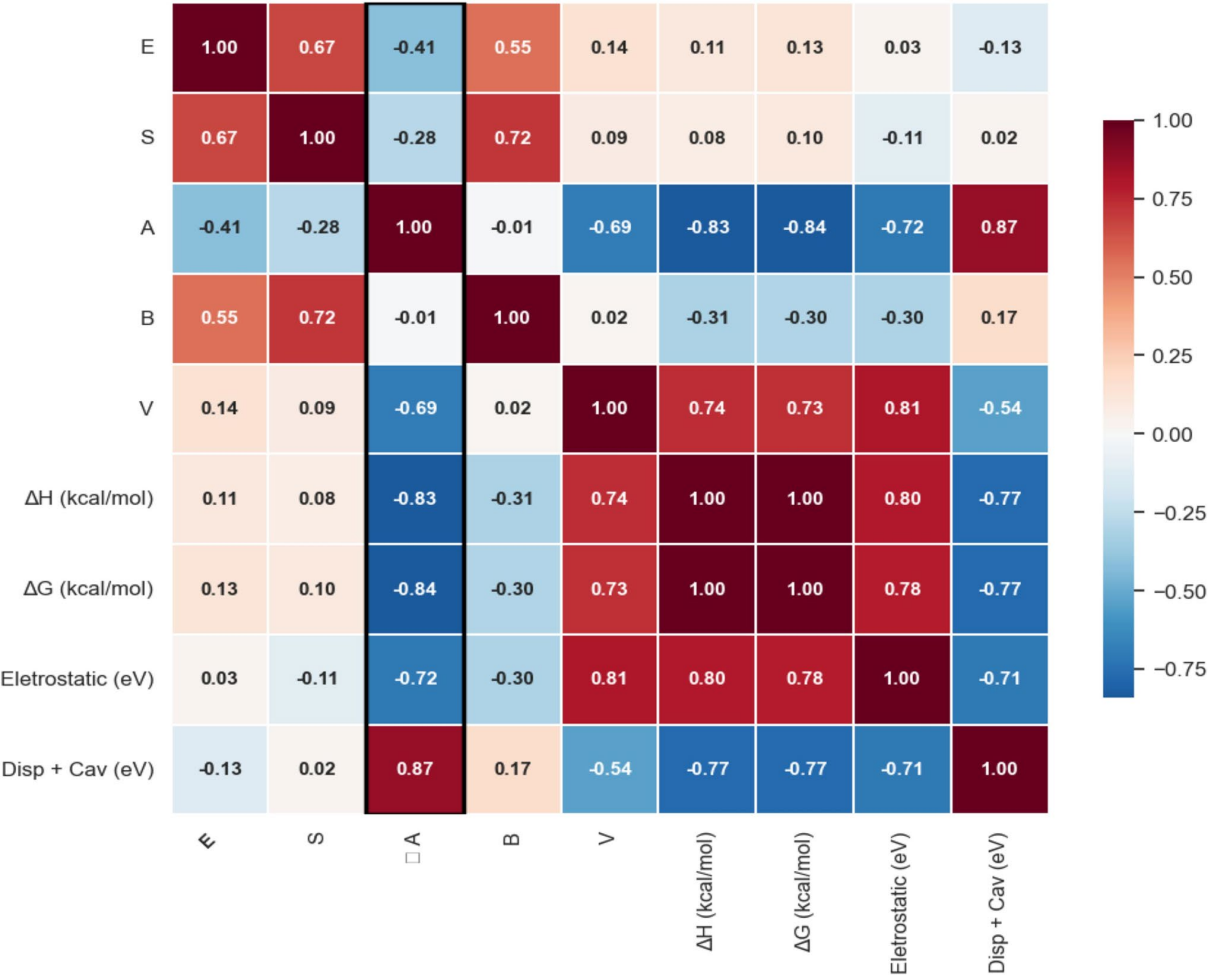
Heatmap depicting the correlations between LFER parameters, thermodynamic properties, and solvation descriptors

*S*_*w*_ is the molar concentration of the solute in water; *c, e, s, a,* and *b* are solvent coefficients; *E* is the solute excess molar refractivity, expressed in units of (cm^3^ mol⁻^1^)/10; *S* represents dipolarity/polarizability of the solute; *A* and *B* denote the aggregate hydrogen-bond acidity and basicity, respectively; and *V* is the McGowan characteristic volume [48]. To clarify the influence of the Abraham parameters, data from the Open Data Database of Compounds with Known Abraham Descriptors, compiled by Bradley et al. [49], were employed. Figure 9 presents a heatmap displaying the correlations between the descriptors of the LFER model and the thermodynamic parameters calculated in the present work.

The SMD model employs several parameters from the Abraham model to describe solvation contributions [31]. Notably, the parameter most closely related to ΔG is *A* (−0.89), which reflects hydrogen-bond acidity, or the capacity to donate hydrogen bonds. This parameter accounts for the stabilizations observed throughout this work, as it significantly impacts the balance between dispersion and cavitation effects. Since the cavitation term is always positive due to the energy required for solvent displacement and solute insertion, increased hydrogen-bonding interactions reduce cavitation energy, given that the cavitation energy depends on the solvent density, which is governed by cohesive interactions. This reduction enhances stabilization, particularly when the solvent is protic. Furthermore, the V parameter, representing McGowan’s characteristic volume, is also strongly correlated with the electrostatic parameter. This indicates that solvents with lower molecular volumes provide greater stabilization of the zwitterionic state. Such findings clarify why methanol stabilizes the zwitterionic state more efficiently than ethanol, and why pyridine exhibits the weakest stabilization among aprotic solvents. Consequently, solvents with smaller molecules tend to exhibit more negative electrostatic values, indicating stronger solute–solvent interactions and enhanced stabilization of the charged zwitterionic structure.

## Conclusion

Amine-based solvents are recognized as an efficient and extensively studied technology for CO_2_ capture. This study investigates how different implicit solvation models describe these reactions. A broad selection of solvents is available to improve both stabilization of the zwitterionic species and efficiency of CO_2_ capture; however, not all solvation models produce reliable results. Models that incorporate more robust intermolecular interaction parameters, such as the SMD model, yield results that are more consistent with experimentally reported data. In this work, we found that PCM, CPCM, and SMD models differ significantly in their predictions of both geometric and thermodynamic parameters associated with the CO_2_ capture, highlighting SMD as the most suitable model for accurately describing this class of reactions.

## Supplementary Information

Below is the link to the electronic supplementary material.

ESM 1 (DOCX 100 KB)

## Data Availability

No datasets were generated or analysed during the current study.
