# Supplementary material for: Implicit solvent effects on the binding interactions of amines with CO2
Source: J Mol Model. 2026 Jul 23;32(8):285. doi: 10.1007/s00894-026-06863-9 (PMC13395902; doi:10.1007/s00894-026-06863-9)
Supplement: Supplementary file 1 — (DOCX 100 KB) [file 894_2026_6863_MOESM1_ESM.docx]

**Supporting Information:**

**Solvent Effects on the Binding Interactions of Amines with CO_2_**

Jonathan B. Brum^a^, José Walkimar de M. Carneiro^a^, Leonardo M. da Costa^a,^*

*^a^Programa de Pós-Graduação em Química, Departamento de Química Inorgânica e Departamento de Química Orgânica, Instituto de Química, Universidade Federal Fluminense. Outeiro de São João Batista s/n 24020-141 Niterói. RJ. Brazil.*

*Corresponding author email:* [*lmcosta@id.uff.br*](mailto:lmcosta@id.uff.br)*;*

Contents

**S1. Geometric data (N–C distance and O–C–O angle) calculated using CAM-B3LYP/6-311++G(d,p) with the PCM, CPCM, and SMD solvation models..73**

**S2. Thermodynamic data (ΔH and ΔG) calculated using CAM-B3LYP/6-311++G(d,p) with the PCM, CPCM, and SMD solvation models. .304**

**S3. Relative stabilization energies of amines and zwitterionic states using hexane as the reference.. .304**

**S1. Geometric data (N–C distance and O–C–O angle) calculated using CAM-B3LYP/6-311++G(d,p) with the PCM, CPCM, and SMD solvation models**

Table 1: Interaction distance (Å) between the NH_2_ amino group and CO_2_ calculated for hexane, acetic acid, trifluoroethanol, dimethyl sulfoxide, water, and formamide using the PCM, CPCM, and SMD solvation models.

| Solvent | PCM | | | | CPCM | | | | SMD | | | |
| --- | --- | --- | --- | --- | --- | --- | --- | --- | --- | --- | --- | --- |
|  | MeNH_2_ | Me_2_NH | Me_3_N | PyNH_2_ | MeNH_2_ | Me_2_NH | Me_3_N | PyNH_2_ | MeNH_2_ | Me_2_NH | Me_3_N | PyNH_2_ |
| Hexane | 2.813 | 2.800 | 2.788 | 1.701 | 2.834 | 2.818 | 2.796 | 1.654 | 2.814 | 2.776 | 2.774 | 1.665 |
| Acetic Acid | 1.708 | 1.657 | 1.741 | 1.584 | 1.636 | 1.618 | 1.674 | 1.564 | 1.593 | 1.581 | 1.624 | 1.535 |
| TFE | 1.649 | 1.623 | 1.681 | 1.559 | 1.623 | 1.609 | 1.661 | 1.556 | 1.583 | 1.573 | 1.614 | 1.528 |
| DCE | 1.673 | 1.639 | 1.707 | 1.571 | 1.618 | 1.605 | 1.656 | 1.553 | 1.617 | 1.607 | 1.660 | 1.561 |
| Py | 1.664 | 1.633 | 1.697 | 1.567 | 1.614 | 1.602 | 1.652 | 1.550 | 1.625 | 1.616 | 1.675 | 1.569 |
| Isopropanol | 1.653 | 1.626 | 1.686 | 1.562 | 1.609 | 1.598 | 1.646 | 1.546 | 1.580 | 1.573 | 1.614 | 1.528 |
| Acetone | 1.652 | 1.626 | 1.685 | 1.561 | 1.607 | 1.600 | 1.644 | 1.545 | 1.613 | 1.607 | 1.660 | 1.560 |
| Ethanol | 1.649 | 1.623 | 1.682 | 1.559 | 1.605 | 1.596 | 1.642 | 1.543 | 1.573 | 1.566 | 1.605 | 1.521 |
| Methanol | 1.646 | 1.621 | 1.678 | 1.558 | 1.603 | 1.594 | 1.640 | 1.542 | 1.566 | 1.560 | 1.597 | 1.513 |
| Nitromethane | 1.644 | 1.620 | 1.677 | 1.557 | 1.603 | 1.593 | 1.639 | 1.541 | 1.607 | 1.600 | 1.652 | 1.555 |
| Ethylene Glycol | --- | --- | --- | --- | 1.603 | 1.593 | 1.639 | 1.541 | 1.565 | 1.558 | 1.595 | 1.512 |
| DMAc | 1.644 | 1.620 | 1.677 | 1.557 | 1.602 | 1.593 | 1.639 | 1.541 | 1.613 | 1.607 | 1.662 | 1.561 |
| DMSO | 1.642 | 1.619 | 1.675 | 1.556 | 1.601 | 1.592 | 1.638 | 1.540 | 1.612 | 1.606 | 1.660 | 1.560 |
| Water | 1.639 | 1.617 | 1.672 | 1.554 | 1.600 | 1.591 | 1.636 | 1.539 | 1.563 | 1.556 | 1.592 | 1.510 |
| FMA | 1.644 | 1.612 | 1.670 | 1.554 | 1.599 | 1.590 | 1.635 | 1.538 | 1.561 | 1.555 | 1.591 | 1.509 |

Table 2: OCO angle (°) of carbon dioxide when interacting with the NH_2_ amino group, calculated for hexane, acetic acid, trifluoroethanol, dimethyl sulfoxide, water, and formamide using the PCM, CPCM, and SMD solvation models.

| Solvent | PCM | | | | CPCM | | | | SMD | | | |
| --- | --- | --- | --- | --- | --- | --- | --- | --- | --- | --- | --- | --- |
|  | MeNH_2_ | Me_2_NH | Me_3_N | PyNH_2_ | MeNH_2_ | Me_2_NH | Me_3_N | PyNH_2_ | MeNH_2_ | Me_2_NH | Me_3_N | PyNH_2_ |
| Hexane | 175.68 | 175.56 | 175.41 | 142.36 | 175.88 | 175.71 | 175.43 | 140.23 | 175.38 | 174.80 | 174.69 | 140.74 |
| Acetic Acid | 141.72 | 139.57 | 141.28 | 136.53 | 139.57 | 137.57 | 138.27 | 135.31 | 136.43 | 135.67 | 135.81 | 133.57 |
| TFE | 139.02 | 137.82 | 138.57 | 135.01 | 137.85 | 137.05 | 137.61 | 134.79 | 135.84 | 135.16 | 135.24 | 133.03 |
| DCE | 140.21 | 138.67 | 139.78 | 139.32 | 137.57 | 136.83 | 137.33 | 134.57 | 137.67 | 137.04 | 137.59 | 135.11 |
| Py | 139.76 | 138.37 | 139.33 | 138.91 | 137.36 | 136.67 | 137.13 | 134.39 | 138.08 | 137.47 | 138.20 | 135.55 |
| Isopropanol | 139.29 | 138.02 | 138.84 | 135.20 | 137.03 | 136.39 | 136.80 | 134.12 | 135.49 | 134.90 | 135.01 | 132.85 |
| Acetone | 139.23 | 137.98 | 138.78 | 135.15 | 136.93 | 136.31 | 136.71 | 134.03 | 137.45 | 136.91 | 137.51 | 135.02 |
| Ethanol | 139.07 | 137.86 | 138.63 | 135.04 | 136.83 | 136.23 | 136.61 | 133.95 | 135.06 | 134.52 | 134.53 | 132.39 |
| Methanol | 138.90 | 137.73 | 138.45 | 134.92 | 136.70 | 136.12 | 136.49 | 133.84 | 134.61 | 134.09 | 134.02 | 131.85 |
| Nitromethane | 138.84 | 137.68 | 138.39 | 134.88 | 136.67 | 136.09 | 136.45 | 133.80 | 137.12 | 136.61 | 137.11 | 134.71 |
| Ethylene Glycol | --- | --- | --- | --- | 136.66 | 136.09 | 136.44 | 133.80 | 134.55 | 134.03 | 133.97 | 131.80 |
| DMAc | 138.83 | 137.67 | 138.38 | 134.87 | 136.65 | 136.08 | 136.44 | 133.79 | 137.41 | 136.92 | 137.53 | 135.03 |
| DMSO | 138.75 | 137.61 | 138.29 | 134.81 | 136.59 | 136.03 | 136.38 | 133.74 | 137.34 | 136.86 | 137.45 | 134.97 |
| Water | 138.59 | 137.49 | 138.13 | 134.70 | 136.47 | 135.93 | 136.27 | 133.64 | 137.34 | 133.77 | 133.66 | 131.55 |
| FMA | 138.54 | 137.44 | 138.07 | 134.70 | 136.43 | 135.90 | 136.22 | 133.60 | 134.36 | 133.87 | 133.79 | 131.61 |

**S2. Thermodynamic data (ΔH and ΔG) calculated using CAM-B3LYP/6-311++G(d,p) with the PCM, CPCM, and SMD solvation models**

Table 3: Enthalpy variation (kcal/mol) for the interaction between the NH_2_ amino group and CO_2_, calculated for hexane, acetic acid, trifluoroethanol, dimethyl sulfoxide, water, and formamide using the PCM, CPCM, and SMD solvation models.

| Solvent | PCM | | | | CPCM | | | | SMD | | | |
| --- | --- | --- | --- | --- | --- | --- | --- | --- | --- | --- | --- | --- |
|  | MeNH_2_ | Me_2_NH | Me_3_N | PyNH_2_ | MeNH_2_ | Me_2_NH | Me_3_N | PyNH_2_ | MeNH_2_ | Me_2_NH | Me_3_N | PyNH_2_ |
| Hexane | 13.30 | 13.26 | 13.29 | 17.54 | -1.47 | -1.54 | -1.59 | 1.52 | -2.15 | -2.34 | -2.35 | 0.66 |
| Acetic Acid | 3.45 | 1.55 | 1.60 | -1.24 | 1.08 | -0.63 | 0.61 | -2.86 | -4.46 | -6.02 | -4.59 | -8.38 |
| TFE | 1.74 | -0.18 | 0.89 | -2.99 | 0.29 | -1.36 | 0.01 | -3.57 | -5.38 | -6.91 | -5.36 | -9.18 |
| DCE | 2.68 | 0.74 | 1.60 | -2.07 | -0.08 | -1.70 | -0.27 | -3.90 | -1.40 | -3.06 | -1.49 | -4.39 |
| Py | 2.37 | 0.43 | 1.37 | -2.39 | -0.38 | -1.97 | -0.50 | -4.16 | -0.98 | -2.71 | -1.16 | -3.81 |
| Isopropanol | 1.98 | 0.05 | 1.07 | -2.77 | -0.85 | -2.40 | -0.86 | -4.58 | -5.51 | -6.77 | -5.08 | -8.63 |
| Acetone | 1.93 | 0.00 | 1.03 | -2.82 | -1.00 | -2.53 | -0.97 | -4.71 | -1.52 | -3.19 | -1.55 | -4.27 |
| Ethanol | 1.79 | -0.14 | 0.92 | -2.95 | -1.15 | -2.67 | -1.08 | -4.84 | -6.59 | -7.89 | -6.08 | -9.75 |
| Methanol | 1.63 | -0.29 | 0.80 | -3.10 | -1.35 | -2.85 | -1.24 | -5.02 | -7.81 | -9.13 | -7.24 | -11.07 |
| Nitromethane | 1.57 | -0.35 | 0.75 | -3.15 | -1.40 | -2.90 | -1.28 | -5.07 | -2.28 | -3.90 | -2.24 | -5.00 |
| Ethylene Glycol | --- | --- | --- | --- | -1.42 | -2.91 | -1.29 | -5.09 | -8.69 | -9.92 | -8.18 | -12.09 |
| DMAc | 0.97 | -0.36 | 0.74 | -3.17 | -1.43 | -2.92 | -1.30 | -5.10 | -1.87 | -3.55 | -1.86 | -4.46 |
| DMSO | 1.53 | -0.45 | 0.66 | -3.25 | -1.53 | -3.02 | -1.38 | -5.19 | -2.13 | -3.84 | -2.16 | -4.72 |
| Water | 1.38 | -0.59 | 0.55 | -3.39 | -1.72 | -3.18 | -1.52 | -5.35 | -6.87 | -7.49 | -5.27 | -10.11 |
| FMA | 1.32 | -0.65 | 0.50 | -3.45 | -1.79 | -3.24 | -1.58 | -5.41 | -9.36 | -10.53 | -8.80 | -12.79 |

Table 4: Gibbs free energy variation (kcal/mol) for the interaction between the NH_2_ amino group and CO_2_, calculated for hexane, acetic acid, trifluoroethanol, dimethyl sulfoxide, water, and formamide using the PCM, CPCM, and SMD solvation models.

| Solvent | PCM | | | | CPCM | | | | SMD | | | |
| --- | --- | --- | --- | --- | --- | --- | --- | --- | --- | --- | --- | --- |
|  | MeNH_2_ | Me_2_NH | Me_3_N | PyNH_2_ | MeNH_2_ | Me_2_NH | Me_3_N | PyNH_2_ | MeNH_2_ | Me_2_NH | Me_3_N | PyNH_2_ |
| Hexane | -11.07 | -10.76 | -12.02 | -3.49 | 6.10 | 6.55 | 6.10 | 12.33 | 5.62 | 6.05 | 6.37 | 11.50 |
| Acetic Acid | 13.01 | 11.37 | 13.23 | 8.94 | 11.60 | 10.42 | 11.96 | 8.50 | 6.18 | 5.08 | 6.93 | 3.15 |
| TFE | 11.18 | 9.80 | 10.69 | 7.33 | 10.87 | 9.71 | 11.41 | 7.83 | 5.20 | 4.22 | 6.20 | 2.39 |
| DCE | 12.33 | 10.63 | 10.30 | 8.18 | 10.53 | 9.39 | 11.15 | 7.52 | 9.25 | 7.92 | 9.89 | 7.07 |
| Py | 12.02 | 10.35 | 10.84 | 7.89 | 10.25 | 9.12 | 10.94 | 7.26 | 9.70 | 8.35 | 10.27 | 7.64 |
| Isopropanol | 11.58 | 10.00 | 10.77 | 7.54 | 9.81 | 8.71 | 10.60 | 6.87 | 5.03 | 4.17 | 6.48 | 2.95 |
| Acetone | 11.50 | 9.96 | 10.76 | 7.50 | 9.67 | 8.58 | 10.50 | 6.74 | 9.23 | 7.85 | 9.85 | 7.22 |
| Ethanol | 11.29 | 9.84 | 10.71 | 7.37 | 9.53 | 8.45 | 10.39 | 6.62 | 4.03 | 3.27 | 5.48 | 1.83 |
| Methanol | 10.56 | 9.71 | 10.63 | 7.23 | 9.34 | 8.27 | 10.25 | 6.45 | 2.84 | 2.07 | 4.38 | 0.56 |
| Nitromethane | 10.02 | 9.66 | 10.61 | 7.18 | 9.29 | 8.22 | 10.21 | 6.40 | 8.44 | 7.20 | 9.16 | 6.52 |
| Ethylene Glycol | --- | --- | --- | --- | 9.27 | 8.21 | 10.19 | 6.38 | 1.96 | 1.30 | 3.42 | -0.46 |
| DMAc | 12.14 | 9.64 | 10.61 | 7.17 | 9.26 | 8.20 | 10.19 | 6.38 | 8.86 | 7.55 | 9.62 | 7.03 |
| DMSO | 11.45 | 9.57 | 10.50 | 7.09 | 9.17 | 8.11 | 10.11 | 6.29 | 8.60 | 7.27 | 9.32 | 6.78 |
| Water | 11.31 | 9.45 | 10.49 | 6.96 | 8.99 | 7.95 | 9.98 | 6.13 | 3.73 | 3.69 | 6.26 | 1.48 |
| FMA | 11.27 | 9.39 | 10.44 | 6.90 | 8.93 | 7.89 | 9.93 | 6.08 | 1.28 | 0.69 | 2.81 | -1.14 |

**S3. Relative stabilization energies of amines and zwitterionic states using hexane as the reference.**

Table 5: Stabilization energies ΔG_norm_ (kcal/mol) of the isolated amine and CO_2_ using hexane as the reference, calculated with the PCM, CPCM, and SMD solvation models.

| Solvent | PCM | | | | CPCM | | | | SMD | | | |
| --- | --- | --- | --- | --- | --- | --- | --- | --- | --- | --- | --- | --- |
|  | MeNH_2_ | Me_2_NH | Me_3_N | PyNH_2_ | MeNH_2_ | Me_2_NH | Me_3_N | PyNH_2_ | MeNH_2_ | Me_2_NH | Me_3_N | PyNH_2_ |
| Acetic Acid | -17.37 | -17.07 | -16.76 | -19.03 | -2.75 | -2.38 | -2.02 | -3.53 | 4.59 | 5.10 | 5.86 | 4.55 |
| TFE | -18.34 | -17.92 | -17.49 | -20.76 | -3.11 | -2.69 | -2.29 | -4.20 | 3.70 | 4.27 | 5.08 | 3.04 |
| DCE | -17.84 | -17.47 | -17.09 | -19.86 | -3.28 | -2.83 | -2.41 | -4.50 | -1.75 | -1.22 | -0.66 | -4.24 |
| Py | -18.01 | -17.63 | -17.25 | -20.17 | -3.40 | -2.94 | -2.50 | -4.74 | -2.95 | -2.30 | -1.70 | -5.35 |
| Isopropanol | -18.22 | -17.81 | -17.40 | -20.54 | -3.60 | -3.12 | -2.64 | -5.12 | -1.08 | -0.45 | 0.29 | -2.80 |
| Acetone | -18.24 | -17.83 | -17.42 | -20.59 | -3.67 | -3.17 | -2.69 | -5.24 | -3.43 | -2.72 | -2.05 | -5.99 |
| Ethanol | -18.32 | -17.90 | -17.48 | -20.72 | -3.73 | -3.22 | -2.73 | -5.35 | -0.56 | 0.08 | 0.86 | -2.34 |
| Methanol | -18.40 | -17.97 | -17.53 | -20.87 | -3.81 | -3.29 | -2.79 | -5.51 | 0.19 | 0.86 | 1.69 | -1.46 |
| Nitromethane | -18.43 | -18.00 | -17.56 | -20.92 | -3.83 | -3.31 | -2.81 | -5.55 | -2.18 | -1.35 | -0.56 | -4.72 |
| Ethylene Glycol | --- | --- | --- | --- | -3.84 | -3.32 | -2.82 | -5.57 | 4.08 | 5.06 | 6.21 | 3.89 |
| DMAc | -18.44 | -18.00 | -17.56 | -20.93 | -3.84 | -3.32 | -2.82 | -5.58 | -3.47 | -2.59 | -1.80 | -5.77 |
| DMSO | -18.48 | -18.04 | -17.59 | -21.01 | -3.89 | -3.36 | -2.85 | -5.65 | -2.78 | -1.73 | -0.79 | -4.65 |
| Water | -18.56 | -18.11 | -17.65 | -21.15 | -3.96 | -3.42 | -2.90 | -5.79 | -0.15 | 0.73 | 1.96 | -1.48 |
| FMA | -18.59 | -18.14 | -17.66 | -21.21 | -3.99 | -3.45 | -2.92 | -5.85 | 5.43 | 6.47 | 7.70 | 5.22 |

Table 6: Stabilization energies ΔG_norm_ (kcal/mol) of the zwitterionic state using hexane as the reference, calculated with the PCM, CPCM, and SMD solvation models.

| Solvent | PCM | | | | CPCM | | | | SMD | | | |
| --- | --- | --- | --- | --- | --- | --- | --- | --- | --- | --- | --- | --- |
|  | MeNH_2_ | Me_2_NH | Me_3_N | PyNH_2_ | MeNH_2_ | Me_2_NH | Me_3_N | PyNH_2_ | MeNH_2_ | Me_2_NH | Me_3_N | PyNH_2_ |
| Acetic Acid | 6.71 | 5.06 | 8.50 | 20.48 | 2.75 | 1.49 | 3.16 | -8.77 | 5.15 | 4.14 | 6.42 | -3.80 |
| TFE | 3.92 | 2.64 | 5.23 | -9.93 | 1.66 | 0.47 | 2.35 | -10.11 | 3.28 | 2.45 | 4.90 | -6.08 |
| DCE | 5.57 | 3.92 | 5.23 | -8.18 | 1.15 | 0.00 | 1.97 | -10.73 | 1.88 | 0.65 | 2.86 | -8.67 |
| Py | 5.08 | 3.48 | 5.62 | -8.78 | 0.75 | -0.37 | 1.66 | -11.22 | 1.13 | 0.01 | 2.19 | -9.20 |
| Isopropanol | 4.44 | 2.95 | 5.39 | -9.50 | 0.11 | -0.96 | 1.18 | -12.00 | -1.67 | -2.33 | 0.40 | -11.35 |
| Acetone | 4.33 | 2.89 | 5.36 | -9.59 | -0.09 | -1.14 | 1.03 | -12.24 | 0.18 | -0.92 | 1.42 | -10.27 |
| Ethanol | 4.05 | 2.70 | 5.25 | -9.85 | -0.30 | -1.33 | 0.88 | -12.48 | -2.15 | -2.70 | -0.03 | -12.01 |
| Methanol | 3.23 | 2.49 | 5.12 | -10.14 | -0.57 | -1.58 | 0.67 | -12.81 | -2.59 | -3.12 | -0.30 | -12.40 |
| Nitromethane | 2.66 | 2.42 | 5.07 | -10.24 | -0.64 | -1.64 | 0.62 | -12.90 | 0.64 | -0.20 | 2.23 | -9.71 |
| Ethylene Glycol | --- | --- | --- | --- | -0.67 | -1.66 | 0.60 | -12.93 | 0.43 | 0.31 | 3.25 | -8.07 |
| DMAc | 4.78 | 2.40 | 5.07 | -10.27 | -0.68 | -1.68 | 0.59 | -12.94 | -0.23 | -1.09 | 1.44 | -10.24 |
| DMSO | 4.04 | 2.29 | 4.93 | -10.43 | -0.82 | -1.80 | 0.48 | -13.11 | 0.20 | -0.51 | 2.16 | -9.37 |
| Water | 3.83 | 2.09 | 4.87 | -10.69 | -1.06 | -2.03 | 0.30 | -13.40 | -2.04 | -1.63 | 1.85 | -11.50 |
| FMA | 3.76 | 2.01 | 4.81 | -10.81 | -1.16 | -2.11 | 0.22 | -13.52 | 1.09 | 1.12 | 4.14 | -7.43 |
